# Conformational Trimorphism in an Ionic Cocrystal of Hesperetin

**DOI:** 10.1021/acs.cgd.2c00861

**Published:** 2022-10-04

**Authors:** Shasha Jin, Molly M. Haskins, Yassin H. Andaloussi, Ruiling Ouyang, Junbo Gong, Michael J. Zaworotko

**Affiliations:** † Department of Chemical Sciences, Bernal Institute, University of Limerick, Limerick V94 T9PX, Ireland; ‡ State Key Laboratory of Chemical Engineering, School of Chemical Engineering and Technology, Tianjin University, Tianjin 300072, People’s Republic of China

## Abstract

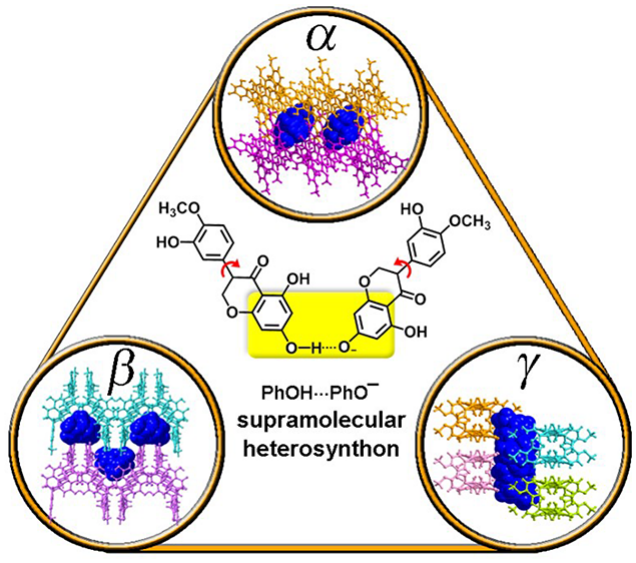

We report the existence of conformational polymorphism in an ionic cocrystal (ICC) of the nutraceutical compound hesperetin (HES) in which its tetraethylammonium (TEA^+^) salt serves as a coformer. Three polymorphs, HESTEA-α, HESTEA-β and HESTEA-γ, were characterized by single-crystal X-ray diffraction (SCXRD). Each polymorph was found to be sustained by phenol···phenolate supramolecular heterosynthons that self-assemble with phenol···phenol supramolecular homosynthons into *C*_3_^2^(7) H-bonded motifs. Conformational variability in HES moieties and different relative orientations of the H-bonded motifs resulted in distinct crystal packing patterns: HESTEA-α and HESTEA-β exhibit H-bonded sheets; HESTEA-γ is sustained by bilayers of H-bonded tapes. All three polymorphs were found to be stable upon exposure to humidity under accelerated stability conditions for 2 weeks. Under competitive slurry conditions, HESTEA-α was observed to transform to the β or γ forms. Solvent selection impacted the relationship between HESTEA-β (favored in EtOH) and HESTEA-γ (favored in MeOH). A mixture of the β and γ forms was found to be present following H_2_O slurry.

## Abstract 2

The phenol···phenolate supramolecular heterosynthon is persistent in three polymorphs of the ionic cocrystal formed by hesperetin and its tetraethylammonium salt.

Crystalline forms of active pharmaceutical ingredients (APIs) are generally the preferred type of solid dosage forms in drug products because of their relative stability, ease of purification, and manufacturability when compared with corresponding amorphous forms.^1^ Crystalline forms of drug molecules and other biologically active compounds, such as nutraceuticals^2−4^ and agrochemicals,^5^ can include polymorphs and multicomponent crystals such as salts, solvates (including hydrates), or cocrystals.^6−13^ Crystalline forms are relevant to oral drug delivery because they can influence physicochemical properties such as solubility and stability.^14^ Solid form screening,^6^ including high-throughput screening,^15^ to identify and characterize crystalline solid forms of drug molecules is therefore a key step during early drug development.^16^ The first step of solid form screening focuses upon identification of which compound should be selected for development, e.g. the neutral drug molecule, a pharmaceutically acceptable salt or a pharmaceutical cocrystal.^17,18^ Generally, the neutral (free acid or free base) form of a drug molecule would be preferred if it has suitable physicochemical properties, but the majority of new chemical entities being developed in the pharmaceutical industry exhibit low solubility,^19,20^ as defined by the Biopharmaceutical Classification System, BCS.^21^ Therefore, since polymorphs and hydrates tend not to offer significant changes in solubility,^22^ pharmaceutical salts^23−25^ and pharmaceutical cocrystals,^26−30^ which involve pharmaceutically acceptable salt or cocrystal formers, respectively, are typically then considered as possible lead candidates. Whereas cocrystals have long been known,^31^ their amenability to design through crystal engineering approaches was not well recognized until the early 2000s when four papers detailed the design of pharmaceutical cocrystals.^32−35^ Successful crystal engineering approaches to cocrystal design are generally based on a knowledge of possible H-bonded supramolecular synthons.^36^ In this context, H-bonded supramolecular heterosynthons^35^ between coformers are key to understanding and designing cocrystals since their hierarchies^37−42^ can be used to project whether a cocrystal is amenable to being readily isolated.^43^ Pharmaceutical cocrystals can significantly diversify the number of crystal forms available for a given API, thereby improving the likelihood that a crystalline form suitable for use in a drug product will be identified. Most importantly, pharmaceutical cocrystals can enhance the solubility of low solubility drug molecules to improve drug product performance.^26,44^ Molecular cocrystals (MCCs), which are cocrystals containing two or more nonvolatile neutral coformers in a stoichiometric ratio, have often been targeted when preparing pharmaceutical cocrystals.^8,45^ Ionic cocrystals^46^ (ICCs) comprise at least one coformer that is a salt. Whereas both classes of cocrystals are typically sustained by H-bonds^47^ or halogen bonds,^48^ ICCs are almost always sustained by charge-assisted H-bonds, which are typically relatively strong in the context of H-bonds.^47^ ICCs can also be based upon coordination bonds.^49,50^

ICCs must have at least three components (cation + anion + neutral or ionic coformer) in the crystal lattice, i.e., A^+^B^–^C, where A^+^ = cation, B^–^ = anion, and C = neutral coformer. ICCs therefore offer at least two variables that can be altered, which increases diversity in terms of composition and, therefore, properties. This contrasts with MCCs, which are typically composed of two molecular coformers, i.e., AB cocrystals. ICCs of general formula A^+^B^–^A or A^+^B^–^B, i.e., ICCs in which a free base or a free acid serves as the coformer with a salt of that base or acid, respectively, are also feasible. Such ICCs are of interest to pharmaceutical science since the active component of the ICC will represent a relatively high mass % of the resulting drug substance, which in turn results in a lower drug dosage. The marketed drug product Depakote is based upon a drug substance that is the ICC of valproic acid and sodium valproate and, therefore, exemplifies A^+^B^–^B drug substances.^51^ Other examples of A^+^B^–^A or A^+^B^–^B ICCs are presented in Table S1.

Investigation of the polymorphic behavior of an API is relevant to drug development since polymorphs can exhibit different physicochemical, mechanical, and biopharmaceutical properties.^52^ This means that regulatory bodies can require polymorphism studies,^53,54^ and there are strong commercial reasons for evaluating polymorphs since, in exceptional circumstances, the unexpected emergence of a more stable lower solubility polymorph can result in negative consequences, as exemplified by ritonavir (Norvir).^55^ Compared to single-component crystals, systematic studies of polymorphism in cocrystals remain largely understudied even though increasing studies of cocrystals means that the number of polymorphic cocrystals has increased in recent years.^56^ In this context, Aitipamula et al. concluded that the percentage of polymorphic cocrystals is comparable to the percentage of polymorphic single-component crystals on the basis of analysis of the Cambridge Structural Database (CSD).^56^ In general, cocrystal polymorphs can be classified into synthon polymorphs,^57^ conformational polymorphs,^58,59^ packing polymorphs, and tautomeric polymorphs.^60^ Sometimes, cocrystal polymorphs may belong to two or more different classes. Most polymorphic studies on cocrystals reported MCCs exhibiting two^61−63^ or three^64,65^ polymorphic forms. There are only a few reports of dimorphic ICCs,^66^ and even fewer that discuss trimorphic ICCs. Indeed, as far as we know, there is just one case of a trimorphic ICC, the ICC of lithium 4-methoxybenzoate with l-proline recently reported by our group.^67^

In this contribution, we focus upon the nutraceutical hesperetin, HES (Scheme 1), which exhibits potentially useful biological properties such as antioxidant, anti-inflammatory, and antitumor activities,^68,69^ but offers relatively low solubility of 1.35 mg·L^–1^^70^ and low bioavailability.^71,72^ HES belongs to the group of natural products known as flavonoids and contains multiple phenolic groups. Phenols are classified as medium strength H-bond donors^38^ and have been established as being able to form supramolecular heterosynthons with H-bond acceptors such as chloride anions,^39^ carboxylate moieties,^40^ and aromatic nitrogen bases.^41^ Recently, we reported a crystal engineering study on ICCs of phenol and substituted phenol derivatives with their conjugate bases, which indicated that the phenol···phenolate (PhOH···PhO^–^) supramolecular heterosynthon is robust and can be relied upon to form cocrystals.^37^ We report herein on the synthesis and characterization of three polymorphs (α, β, γ) of the A^+^B^–^B type ICC formed between HES and its tetraethylammonium (TEA^+^) salt, HESTEA.

**Scheme 1:**
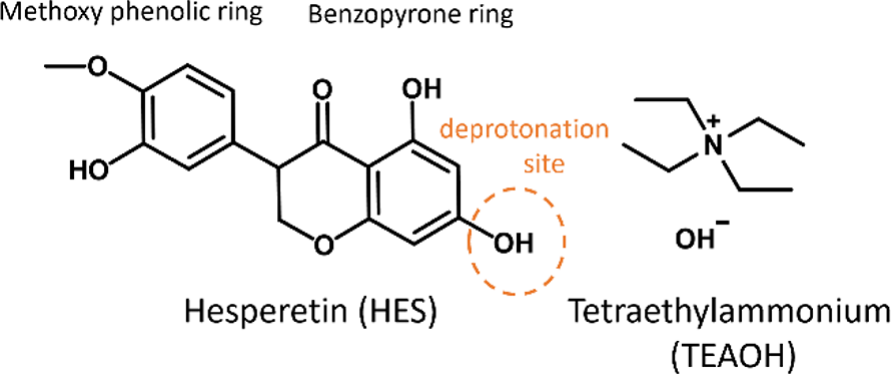
Molecular Structures and Abbreviations for the Coformers Used Herein

HESTEA was prepared by slurrying 150 mg (0.50 mmol) of HES and 186.6 μL (0.25 mmol) of 1.34 M tetraethylammonium hydroxide (TEAOH) in MeOH in 1 mL of MeOH, EtOH, or H_2_O for 24 h. Recrystallization of the resulting bulk powder (shown to be HESTEA-α as determined by powder X-ray diffraction, PXRD) from MeOH via slow evaporation at room temperature afforded single crystals of HESTEA-α, as confirmed by single-crystal X-ray diffraction (SCXRD, see Supporting Information for experimental details). Liquid diffusion involving 1 mL of an EtOH solution of HESTEA powder layered below 2.3 mL of *n*-hexane yielded single crystals of HESTEA-β. HESTEA-γ was isolated by slow evaporation of 0.75 mL of an EtOH solution of 15 mg (0.050 mmol) of HES and 83.3 μL (0.025 mmol) of TEAOH in MeOH diluted to 0.3 M at RT. All three polymorphs were observed to be colorless and formed block-shaped crystals; relevant crystallographic parameters are presented in Table 1. Geometric parameters of the PhOH···PhO̅ and PhOH···PhOH H-bonds in each polymorph are given in Table S2.

**Table 1:** Selected Crystallographic Data and Structure Refinement Parameters

compound abbreviation	HESTEA-α (TEA^+^HES^–^)HES, α	HESTEA-β (TEA^+^HES^–^)HES, β	HESTEA-γ (TEA^+^HES^–^)HES, γ
formula	C_40_H_47_NO_12_		
crystal system	monoclinic	monoclinic	monoclinic
space group	*P*2_1_/*n*	*P*2_1_/*c*	*C*2/*c*
*a* (Å)	14.1323(5)	14.7566(9)	22.7154(7)
*b* (Å)	17.9440(7)	14.9419(10)	8.6532(3)
*c* (Å)	14.5214(4)	16.5652(11)	18.5287(6)
α (deg)	90	90	90
β (deg)	95.486(3)	95.328(2)	90.987(1)
γ (deg)	90	90	90
vol (Å^3^)	3665.6(2)	3636.7(4)	3641.5(2)
density (g·cm^3^)	1.328	1.343	1.336
*Z*, *Z*′	4, 1	4, 1	4, 0.5
*T* (K)	135	135	135
*R*_1_	0.0770	0.0450	0.0520
w*R*_2_	0.1910	0.1142	0.1318
GooF	1.028	1.056	1.134
CCDC	2189972	2189975	2189977

HES crystallized in the space group *P*2_1_/*c* with one molecule in the asymmetric unit. The crystal packing diagram is displayed in Figure S1, which reveals a chain of HES molecules connected through O–H···O=C H-bonds [2.6911(68) Å].^73^

HESTEA-α crystallized in the space group *P*2_1_/*n* with an asymmetric unit comprising one TEA^+^ cation, one HES^–^ anion, and one HES molecule. The phenolate moiety of the HES^–^ anion forms H-bonds to a phenolic group on the benzopyrone ring from an adjacent HES molecule and a phenolic group on the methoxy phenolic ring from an HES^–^ anion via charge-assisted PhOH···PhO^–^ H-bonds [O6···O7^–^, 2.446(3) Å; O11···O7^–^, 2.711(3) Å, respectively]. Additionally, an HES molecule interacts with a phenolic group of a neighboring HES molecule via PhOH···PhOH H-bond [O2···O6, 2.638(3) Å], which results in a *C*_3_^2^(7) H-bonded motif comprising one phenolate and three phenolic groups from two HES^–^ anions and two HES molecules, as illustrated in Figure 1a. When viewed down the *c*-axis, the *C*_3_^2^(7) H-bonded motifs are organized in a “cross” shape and serve as nodes that are cross-linked by HES molecules and HES^–^ anions into H-bonded sheets. In these sheets, HES molecules form helical chains around 2-fold screw axes through PhOH···PhOH H-bonds that propagate along the *b*-axis while HES^–^ anions form zigzag chains through PhOH···PhO^–^ H-bonds that propagate along the *a*-axis (Figure 1b). HES or HES^–^ moieties from adjacent HES or HES^–^ chains align parallel between benzopyrone rings (highlighted in Figure 1b). Adjacent sheets stack via weak H-bonds in such a manner that square-pockets are generated, which contain TEA^+^ cations that are positioned via C–H···O and columbic forces (Figure 1c).

**Figure 1:**
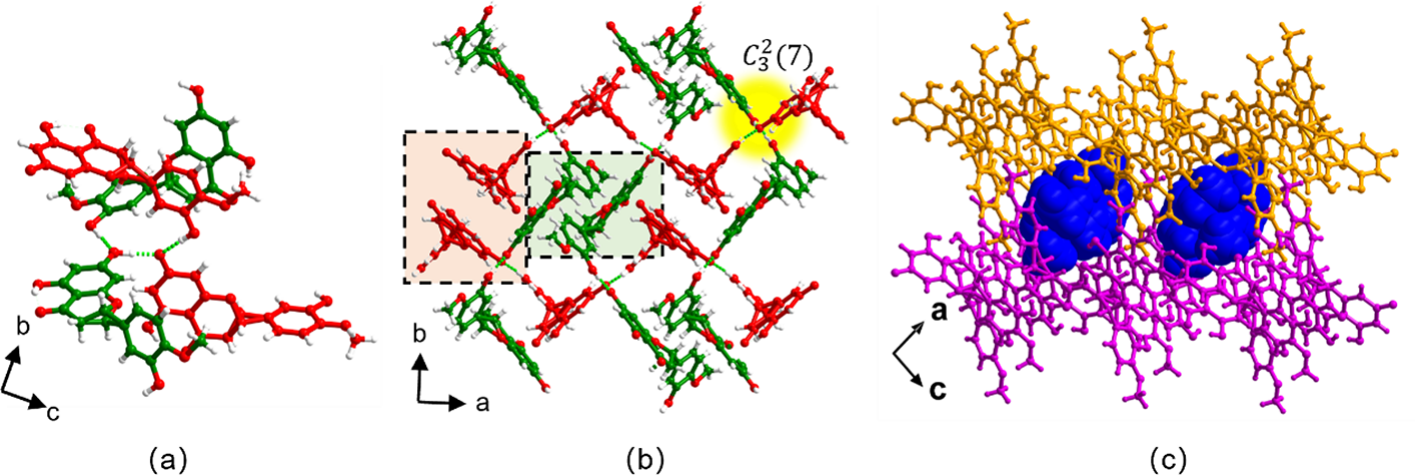
Crystal structure of HESTEA*-α.* HES molecules and HES^–^ anions are colored green and red, respectively. (a) The *C*_3_^2^(7) H-bonded motif formed between phenolic and phenolate groups in HESTEA*-α*. (b) H-bonded sheets with HES moieties organized in zizag chains along the *a* and *b* axes. (c) TEA^+^ cations (blue, space-filling mode) lie in pockets between H-bonded sheets. Cations are omitted for clarity in (a) and (b).

HESTEA*-β* crystallized in the space group *P*2_1_/*c* with an asymmetric unit containing one TEA^+^ cation, one HES^–^ anion and one HES molecule. HES molecules and HES^–^ anions alternate and form a helical chain around a 2-fold screw axis sustained by PhOH···PhO^–^ [O2···O7̅, 2.6573(18) Å] and PhOH···PhOH [O11···O6, 2.6764(18) Å] H-bonds, which propagate along the *b*-axis (Figure 2a). Adjacent helical chains interdigitate via PhOH···PhO^–^ [O6···O7̅, 2.4492(17) Å] H-bonds in such a manner that pairs of HES^–^ or HES are aligned antiparallel (Figure 2a). Like HESTEA*-α, C*_3_^2^(7) H-bonded motifs are formed and comprise two PhOH···PhO^–^ and one PhOH···PhOH H-bonds between one phenolate and three phenolic groups. When viewed down the *a*-axis, the H-bonded motifs are once again organized in a “cross” shape, but HES and HES^–^ adopt a different orientation to that observed in HESTEA*-α.* When viewed down the *c*-axis, adjacent H-bonded sheets interdigitate with each other through weak H-bonds and form cavities containing TEA^+^ cations that are engaged in C–H···O and columbic forces (Figure 2c).

**Figure 2:**
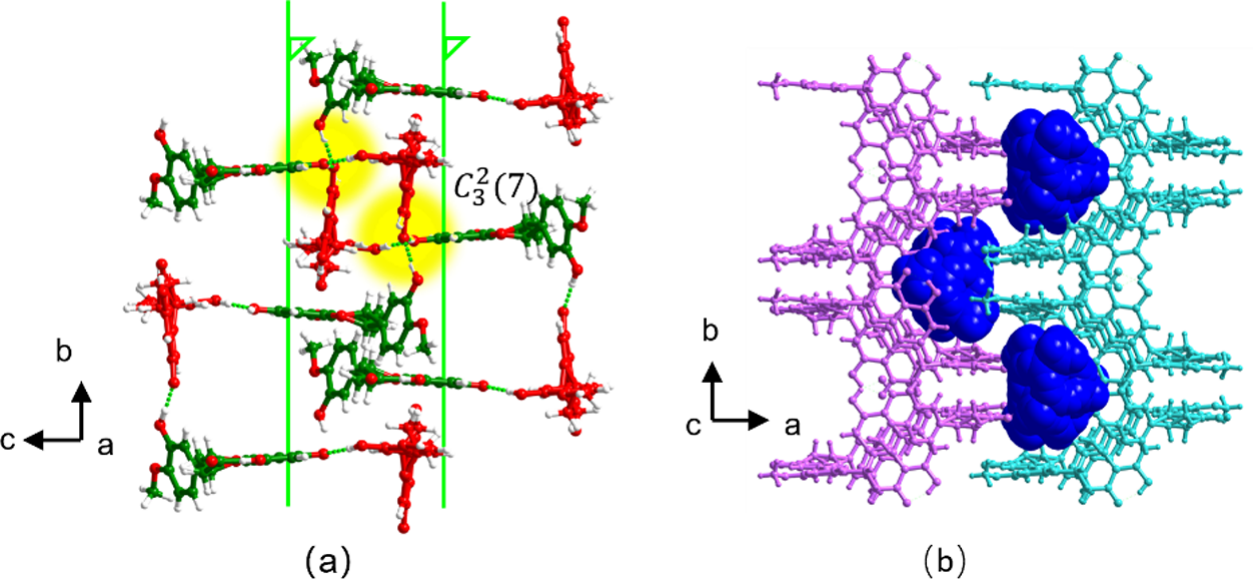
Crystal structure of HESTEA*-β.* HES molecules and HES^–^ anions are colored green and red, respectively. (a) Two helical chains around 2-fold screw axes interdigitate, thereby causing the formation of *C*_3_^2^(7) H-bonded motifs (highlighted in yellow) sustained by two PhOH···PhO^–^ and one PhOH···PhOH H-bonds. Cations are omitted for clarity. (b) TEA^+^ cations (blue, space-filling mode) lie between H-bonded sheets.

HESTEA*-γ* crystallized in the space group *C*2/*c* with an asymmetric unit comprising 0.5 of the chemical formula since TEA^+^ cations are disordered around an inversion center and an apparently symmetric H-bond between HES moieties enables the proton to sit at or close to a crystallographic 2-fold axis. The symmetric or close-to-symmetric nature of the [PhO···H···PhO^–^] anions is supported by the short O6···O6′ distance of 2.4256(19) Å^74^ and location of the proton from difference Fourier map inspection. In our recent work,^37^ a CSD survey revealed that the average O···O^–^ distance for PhOH···PhO^–^ H-bonds is 2.528 ± 0.08 Å; the PhOH···PhO^–^ H-bond in HESTEA*-γ* is shorter than the vast majority of previously reported structures. The classification of this PhOH···PhO^–^ H-bond as symmetric or close-to-symmetric cannot be asserted using SCXRD, and further studies will be required in this context. As illustrated in Figure 3a, [PhO···H···PhO^–^] anions form H-bonds with two phenolic groups on the methoxy-substituted phenolic rings from two neighboring HES moieties [O2···O6, 2.663(2) Å], which forms a *C*_3_^2^(7) motif (Figure 3a). In the H-bonded motifs, HES moieties align in an antiparallel face-to-face arrangement (4.363 Å between two benzopyrone rings), thereby forming a bilayer of tapes along the *c*-axis (Figure 3b). The bilayer of tapes stack around 2-fold rotation axes. The distance of adjacent tapes along the *b*-axis is 4.019 Å (between benzopyrone rings), while along the *c*-axis, TEA^+^ cations lie between adjacent tapes engaged in C–H···O and columbic forces (Figure 3c).

**Figure 3:**
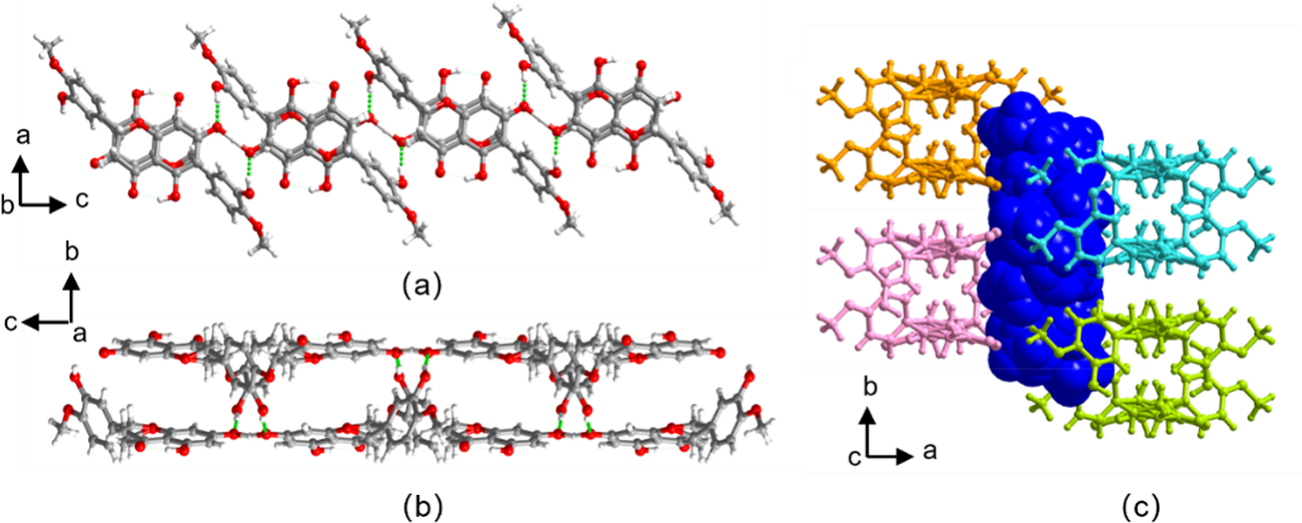
Crystal structure of HESTEA*-γ.* (a) *C*_3_^2^(7) H-bonded motifs formed between phenolic and phenolate groups in HESTEA*-γ* assemble into a bilayer tape along the *c-*axis, as shown in (a) and (b) (cations omitted for clarity). (c) TEA^+^ cations (blue, space-filling mode) lie between the H-bonded tapes.

The experimental PXRD patterns of the HESTEA polymorphs are distinct from each other and match well with the corresponding calculated PXRD patterns (Figure S2). The relative orientation of the two HES moieties in the asymmetric units in HESTEA*-α*, -β, and -γ (Figure 4a–c) can be used to illustrate their different crystal packing patterns. HES molecules in HESTEA*-α* and HESTEA-β are arranged perpendicular in relation to the corresponding HES^–^ anions, with dihedral angles (measured between benzopyrone ring planes from HES and HES^–^) of 88.94° and 76.92°, respectively. In HESTEA*-γ* the HES moieties are closer to planarity with a dihedral angle between two benzopyrone rings of 30.09°. Overall, the *C*_3_^2^(7) H-bonded motifs in the three polymorphs (Scheme 2) differ in the relative orientation of the HES molecules and HES^–^ anions. Conformational differences within the HES moieties become evident when their structures are overlaid (Figure 4d). As seen by aligning the methoxy phenolic moieties, the benzopyrone rings exhibit a high degree of torsional variability, but disorder of the chiral carbons means that torsion angles cannot be readily determined. In general, the conformational variability of HES moieties can be assessed through determination of the dihedral angles between the benzopyrone rings (chiral carbons excluded) and the methoxy phenolic rings of nonequivalent HES moieties. The equivalent dihedral angles of the seven HES entries in the CSD and the three HESTEA polymorphs reported herein are tabulated in Table S3. In HES hydrate (FOYTOC),^75^ the benzopyrone ring and the methoxy phenolic ring are almost parallel (3.69°). In HES cocrystals, HES moieties exhibit dihedral angles that range from 4.69° to 89.06°. This conformational variability is reflected in the HESTEA ICC polymorphs reported herein, which may be classified as conformational polymorphs.^58,60^

**Figure 4:**
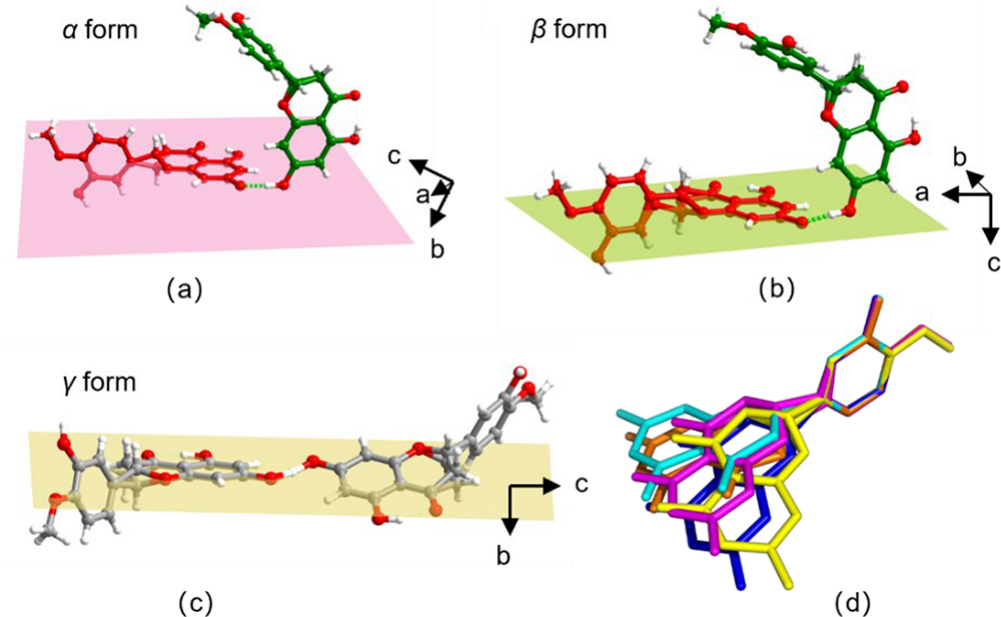
Relative orientation of HES moieties in the asymmetric units of (a) HESTEA*-α*, (b) HESTEA*-β* (HES molecules and anions are colored green and red, respectively), and (c) HESTEA*-γ*. (d) Overlay of the conformations of HES moieties in HESTEA*-α* (yellow for HES, blue for HES^–^), HESTEA*-β* (cyan for HES molecule, magenta for HES^–^), and HESTEA*-γ* (orange). Hydrogen atoms are omitted for clarity. Virtual planes through atoms on the benzopyrone rings are created in (a), (b), and (c). Chiral carbons are not included in plane calculations because they are disordered and nonplanar with other carbon atoms on the benzopyrone rings.

**Scheme 2:**
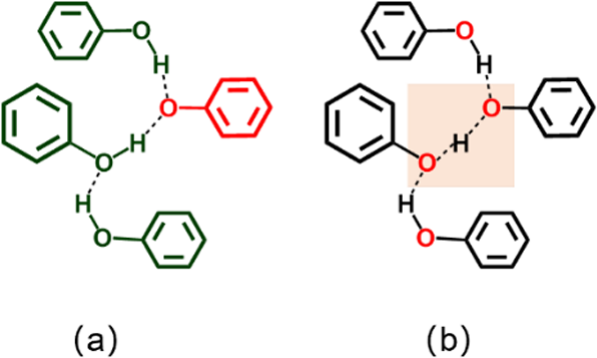
Schematic Representation of (a) the *C*_3_^2^(7) H-Bonded Motif Sustained By PhOH···PhO^–^ and PhOH···PhOH H-Bonds (Phenol, Green; Phenolate, Red) in HESTEA-α and -β and (b) the *C*_3_^2^(7) H-Bonded Motif Comprising Symmetric H-Bond Found in HESTEA-γ

The thermal properties of the three HESTEA polymorphs were investigated by means of thermogravimetric analysis (TGA), differential scanning calorimetry (DSC), and variable temperature powder X-ray diffraction (vt-PXRD). TGA data revealed that HESTEA*-α*, -β, and -γ each decomposed at ca. 240 °C, slightly lower than pure HES, which decomposed at ca. 260 °C (Figure S3a). The DSC curve (Figure S3b) of HESTEA*-α* displayed two endothermal events, the first being consistent with transformation to a new phase at ca. 182 °C followed by a larger endotherm at ca. 193 °C, which we attribute to melting. vt-PXRD data (Figure 5) also reveals a phase change by 180 °C to a new crystal form with a different PXRD to that of any of the three polymorphs characterized by SCXRD. In contrast, heating of HESTEA*-β* and HESTEA-γ resulted in sharp melting endotherms at ca. 192 and 184 °C, respectively, with vt-PXRD indicating that the β and γ polymorphs retained their structures until melting (Figure S4). All three HESTEA polymorphs exhibit lower melting points than pure HES, which exhibited a single sharp melting endotherm at 232 °C.

**Figure 5:**
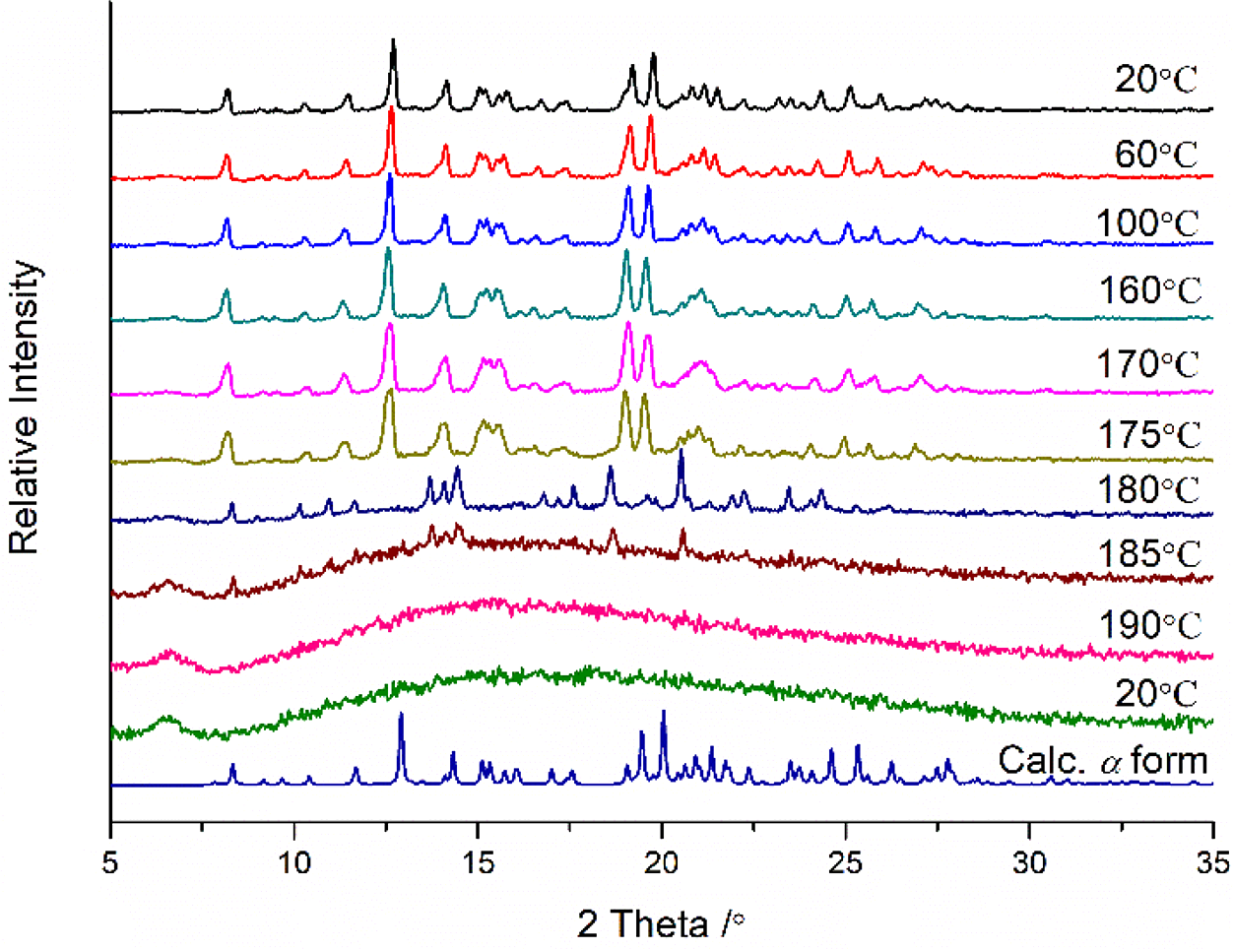
Variable temperature PXRD of HESTEA*-α* (there is peak shift due to thermal expansion as ICC were collected at 135 K).

We also studied the relative stability of the HESTEA polymorphs. When subjected to accelerated stability-testing conditions (40 °C, 75% RH)^76^ for 14 days, all three polymorphs retained stability (Figure S5). HESTEA*-α* was initially obtained in bulk by slurrying as described above. However, after HESTEA*-β* and HESTEA-γ were isolated, HESTEA*-α* could not isolate using the same synthetic conditions. Rather, HESTEA*-β* was thereafter obtained via slurrying in EtOH, and HESTEA*-γ* through slurrying in MeOH or H_2_O (see Supporting Information for details). These slurry experiments suggest that HESTEA*-α* is less stable than β and γ.^77,78^Table 2 and Figure S6 detail the relative stability of HESTEA polymorphs as determined by competitive slurrying of 1:1 mixtures of the α and β polymorphs, α and γ polymorphs, or β and γ polymorphs conducted in 1.5 mL of H_2_O, MeOH, or EtOH. These competitive slurry experiments revealed that HESTEA*-α* transformed into one of the other polymorphs in all three solvents. HESTEA*-β* was found to be stable in EtOH, whereas HESTEA*-γ* was isolated from MeOH, which correlates with the results of slurry synthesis. In the case of H_2_O, even after 2 weeks the resulting powder remained a mixture. That HESTEA*-α* is least stable is also suggested by its lower density^79,80^ (1.328 g·cm^–3^ at 135 K) versus β (1.343 g·cm^–3^) and γ (1.336 g·cm^–3^). HESTEA*-α* might be classified as a disappearing polymorph^77,78^ since we have been unable to make it again despite repeated attempts (see experimental section of Supporting Information for details).

**Table 2:** Results of Competitive Slurry

solvent	α + β	β + γ	α + γ
H_2_O	β	γ + β	γ
MeOH	β	γ	γ
EtOH	β	β	γ

In conclusion, cocrystallization of HES and TEAOH afforded three polymorphs of the new ICC HESTEA. SCXRD revealed that all polymorphs are sustained by PhOH···PhO^–^ and PhOH···PhOH H-bonds that assemble into *C*_3_^2^(7) H-bonded motifs. We attribute the differences in crystal packing to conformational polymorphism. Competitive slurry experiments revealed the relative stability of HESTEA polymorphs in an aqueous environment. The present study confirms the potential to apply crystal engineering to generate ICCs of phenolic compounds sustained by the PhOH···PhO^–^ supramolecular heterosynthon, which is persistent even when multiple polymorphs are possible since the polymorphism in HESTEA can be attributed to conformational differences rather than different H-bonded motifs.
