# Supplementary material for: Conformational Trimorphism in an Ionic Cocrystal of Hesperetin
Source: Cryst Growth Des. 2022 Oct 4;22(11):6390–7. doi: 10.1021/acs.cgd.2c00861 (PMC9634790; doi:10.1021/acs.cgd.2c00861)
Supplement: Supplementary file 1 — cg2c00861_si_001.pdf [file cg2c00861_si_001.pdf]

Supporting Information

Conformational trimorphism in an ionic cocrystal of hesperetin

Shasha Jin,[a] *Molly M. Haskins,*[a] *Yassin H. Andoloussi,*[a] *Ruiling Ouyang,*[b] *Junbo Gong*[b,]* *and Michael J. Zaworotko*[a,]*

[a.] Department of Chemical Sciences and Bernal Institute, University of Limerick, Limerick, V94 T9PX, Ireland

[b.] State Key Laboratory of Chemical Engineering, School of Chemical Engineering and Technology, Tianjin University, Tianjin 300072, People's Republic of China

* Corresponding author: junbo_gong@tju.edu.cn, xtal@ul.ie

Table of Contents

ICCs examples --1
Experimental section ---2,3
Characterization techniques --3,4
HES crystal packing --4
PXRD comparison --5
Geometrical parameters of selected H-bonds -- 5
Dihedral angles of HES in reported crystal structures ------------------------------------6
TGA and DSC plots ---6
Variable temperature PXRD pattern ---7
Results of accelerated stability test --- 7-9
Results of competitive slurry ---9,10
Reference --- 11

Table S1. Examples of ICCs in A^+B^-A or A^+B^-B.

ICC systems	Composition
**A^+B^-B**	
Bumetanide (BUM)<br>Cytosine (CYT)	$BUM^-BUM\cdot CYTH^+$ [1]
Bumetanide (BUM)<br>5-fluoro-cytosine (5FCYT)	$BUM^-BUM\cdot 5FCYTH^+$ [2]
Pefloxacin (PEF)<br>Fumaric acid (FA)	PEF^+FA^-FA [3]
Pefloxacin (PEF)<br>Oxalic acid (OA)	PEF^+OA^-OA [3]
Pefloxacin (PEF)<br>Glutaric acid (GLA)	PEF^+GLA^-GLA [3]
Valproate semisodium (Na^+ VAL^-)<br>Valproic acid (VAL)	$VAL\cdot Na^+\ VAL^-$ [4]
Naproxate sodium (Na^+ NAP^-)<br>Naproxen (NAP)	$NAP\cdot Na^+\ NAP^-$ [5]
Benzoate ammonium ($BEN^-NH_4^+$)<br>Benzoic acid (BEN)	$BEN\cdot NH_4^+BEN^-$ [6]
Benzoate sodium (BEN^-Na^+)<br>Benzoic acid (BEN)V	$2BEN\cdot Na^+BEN^-$ [6]
Benzoate potassium (BEN^-K^+)<br>Benzoic acid (BEN)	$BEN\cdot K^+BEN^-$ [6]
Indomethacin (IND)<br>Lidocaine (LID)	$IND\cdot LID^+IND^-$ [7]
Aspirin (ASP)<br>Aspirinate sodium (ASP^-Na^+)	$(ASP\cdot ASP)^-Na^+$ [8]
Chrysin (ChrH)<br>Berberine (BerbOH)	$Berb^+Chr^-\cdot 2ChrH$ [9]
Escitalopram (ESC)<br>Oxalic acid (OA)	$2ESC^+OA^{2-}OA\cdot 0.325H_2O$ [10]
**A^+B^-A**	
4-aminosalicylic acid (PAS)<br>Cytosine (CYT)	$CYT^+PAS^-CYT\cdot H_2O$ [11]
Nicotinamide (NICA)<br>Nicotinamidium hydrochloride (NICAHCl)	$NICA\cdot NICAH^+Cl^-$ [5]

Experiments

Synthesis. Hesperetin (HES) (analytical standard) and 25% (1.34M) tetraethylammonium hydroxide (TEAOH) in MeOH were purchased from Sigma-Aldrich and used in experiments at the University of Limerick, Ireland. HES (97%) and 25% TEAOH in MeOH (AR) purchased from Aladdin were used in experiments at Tianjin University, China. All solvents were purchased from Sigma-Aldrich and used without further purification.

Single crystals of HESTEA polymorphs. Single crystals of polymorphs were obtained via slow evaporation for HESTEA-*α* and -*γ* and liquid diffusion for HESTEA-*β*. HESTEA was first prepared by slurrying 150 mg (0.50 mmol) of HES and 186.6 µL (0.25 mmol) of 1.34 M TEAOH in MeOH in 1 mL of MeOH, EtOH or H_2O for 24h. Recrystallizing the resulting bulk powder from MeOH via slow evaporation at room temperature afforded single crystals of HESTEA-α as confirmed by Single-Crystal X-ray Diffraction (SCXRD, see SI for experimental details). Liquid diffusion of 1 mL of an EtOH solution of bulk HESTEA powder layered below 2.3 mL of n-hexane yielded single crystals of HESTEA-*β*. HESTEA-*γ* was isolated by slow evaporation of 0.75 ml of an EtOH solution of 15 mg (0.05 mmol) of HES and 83.3 µL TEAOH in MeOH (diluted to 0.3M, 0.025 mmol) at RT.

Bulk preparation of HESTEA polymorphs. HESTEA-*α*, -*β* and -*γ* were scaled up by slurry of 150 mg HES and 186.6 µL TEAOH MeOH solution (1.34M) in a 2:1 mole ratio for 48h in different solvents at RT.

The study of solution formation of HESTEA commenced at Tianjin University, China, in February 2021. Slurry of HES and TEA in H_2O, MeOH and EtOH yielded HESTEA-*α* as confirmed by later SCXRD characterization. The following week we repeated these experiments and unexpectedly obtained HESTEA-*β* in MeOH and EtOH. HESTEA-*α* was still obtained from H_2O over two months. In June 2021, we attempted the synthesis of HESTEA-*α* once again under identical conditions via slurry in H_2O. A mixture of HESTEA-*α* and third new form (HESTEA-*γ*) was obtained. Numerous attempts to synthesize pure HESTEA-*α* using same slurry method under the same conditions failed and no evidence of HESTEA-*α* from PXRD analysis could be found subsequently. Pure HESTEA-*γ* was then generated in H_2O repeatedly in subsequent preparation.

When same slurry experiments were performed in parallel experiments at the University of Limerick, Ireland, a similar phenomenon appeared. In March 2021, pure HESTEA-*α* was obtained from EtOH by one of the authors, YH. HESTEA-*γ* in H_2O and MeOH was also synthesized. In May 2021, one of the authors, SJ moved from Tianjin University to the University of Limerick and she repeated the EtOH slurry, resulting in HESTEA-*β*, at which point HESTEA-*α* had 'disappeared'. These experiment results are consistent with other reported polymorph 'disappearances'. As noted in Bernstein's 1995 review 'it is always possible to obtain 'the old form' again; it is only a matter of finding the right experimental conditions.' Frustratingly, we have not been able to prepare HESTEA-*α* again despite trying using seeding, mechanochemistry and different solvents systems.

Table S2. Slurry results when experiments were done with starting materials from different sources.

Molar ratio HES:TEAOH	Solvents	China	China (after 4 months)	Ireland (materials from China)	Ireland (materials from Europe)
2:1	H_2O	*α*	*α* and *γ*<br>*γ*	*γ*	*γ*
2:1	MeOH	(*α* once)<br>*β*	*β*	*β* and/or *γ*	*γ*
2:1	EtOH	(*α* once)<br>*β*		*β* and/or *γ*	(*α* once)<br>*β*

Accelerated Stability studies. Qualitative stability studies were conducted for HESTEA-*α*, -*β* and -*γ* at 40 °C and 75% RH. The three samples were stored for 14 days and analysed by PXRD at regular time intervals.

Competitive Stability studies. 1:1 mixture of HESTEA-*α* and -*β*, HESTEA-*β* and -*γ*, or HESTEA-*α* and -*γ* were added to 1.5 mL H_2O, MeOH and EtOH, respectively, and then slurried under ambient conditions for one day to two weeks. The solid was then filtered and tested PXRD.

Single-Crystal X-ray Data Collection and Structure Determination. Crystal crystals of three polymorphs were determined by single crystal X-ray diffraction (SCXRD) with either Mo K*α* (λ= 0.7107 Å) radiation for HESTEA-*α* or Cu K*α* (λ=1.5418 Å) for -*β* and -*γ*. Crystallographic data of HESTEA-*α* was collected via Rigaku MicroMax-007 diffractometer equipped with a Raxis IV++ image plate detector and nitrogen-flow Oxford Cryosystem 800. Crystallographic data of HESTEA-*β* and -*γ* were collected via Bruker D8 Quest fixed-chi diffractometer equipped with Photon 100 detector and the nitrogen-flow Oxford Cryosystem attachment. Unit-cell determination, data reduction and absorption correction (multiscan method) were conducted using the Bruker APEX3 suite with implemented SADABS software. All structures were solved using SHELXT and refined using SHELXL contained in Olex2.

Powder X-ray Diffraction (PXRD). All PXRD data were collected on Empyrean diffractometer (PANalytical, philips) with experimental parameters as follows: CuK*α* radiation (λ_α = 1.54056 Å); 40 kV and 40 mA; scan speed 8°/min; step size 0.05°.

In-situ Variable Temperature Powder X-ray Diffraction (VT-PXRD). Diffractograms at different temperature were recorded using a PANalytical X'Pert Pro-MPD diffractometer equipped with a PIXcel3D detector operating in scanning line detector mode with an active length of 4 utilizing 255 channels. Anton Paar TTK 450 stage coupled with the Anton Paar TCU 110 Temperature Control Unit was used to record the variable temperature diffractograms. The diffractometer is outfitted with and Empyrean Cu LEF (long fine-focus) HR (9430 033 7300x) tube operated at 40 kV and 40 mA; and Cu K*α* radiation (λ_α = 1.54056 Å). Continuous scanning mode with the goniometer in the theta-theta orientation was used to collect the data. In experiment,

HESTEA-*α*, -*β* an -*γ* powder were loaded on a zero-background sample holder made for Anton Paar TTK 450 chamber. The data was collected from 5° to 40° with a step size of 0.0334225 and a scan time of 50.165 seconds per step. The PXRD data was collected at designated temperature points, i.e. 20°C, 60°C, 100°C, 160°C, 170°C, 175°C, 180°C, 185°C, 190°C and then cooling to 20°C again at the room temperature and under N_2 atmosphere.

Differential Scanning Calorimetry (DSC). Thermal analysis was conducted using a TA Q2000 heat flux DSC. Microcrystalline samples of three polymorphs were sealed in aluminum pans and a ramp rate of 10 °C/min from ambient temperature to 260 °C was applied under a N_2 atm. Measured heat flow was compared with that of empty aluminium pan.

Thermogravimetric Analyses (TGA). Thermal analysis was performed on a TA Instrument Q50 TG in the range of ambient temperature to 350 °C with a ramp rate of 10 °C/min.

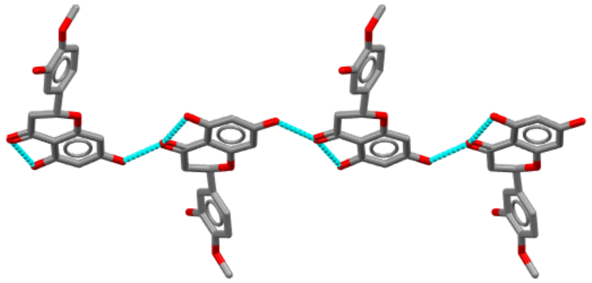

Figure S1. Crystal packing observed in pure HES structure.

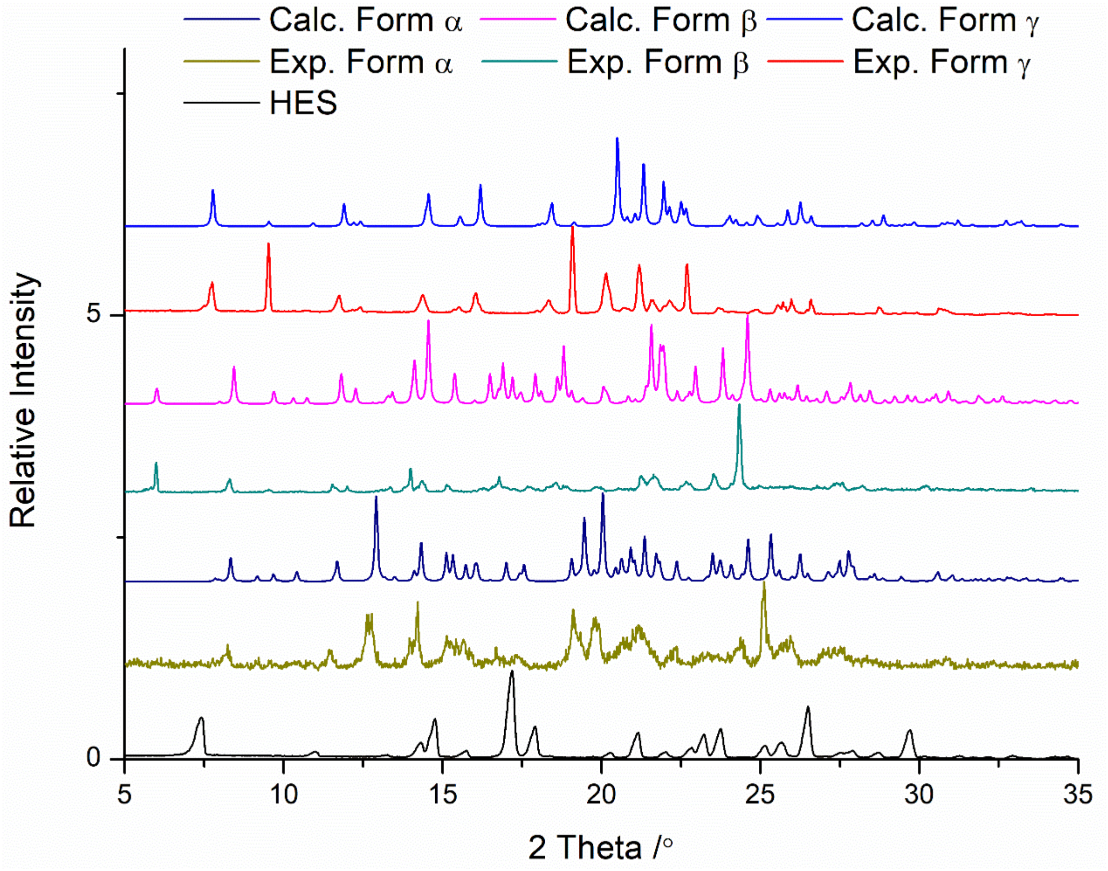

Figure S2. Calculated PXRD generated from single crystal structures of HESTEA-*α*, -*β* and -*γ* compared with experimental PXRD (there is peak shift due to thermal expansion as ICCs were collected at 100K).

Table S3. Hydrogen bonds geometrical parameters in HESTEA-*α*, -*β* and -*γ*.

ICC polymorphs	d (D-H)/Å	d (H···A)/Å	D (D··· A)/Å	θ/°
	0.86(3)	1.78(3)	2.638(3)	176(2)
HESTEA-*α*	0.895(16)	1.552(15)	2.446(3)	179(4)
	0.85(3)	1.87(3)	2.711(3)	170(3)
	0.87(2)	1.79(2)	2.6573(18)	175(2)
HESTEA-*β*	0.92(2)	1.53(2)	2.4492(17)	174(3)
	0.86(2)	1.82(2)	2.6764(18)	178(3)
HESTEA-*γ*	0.91(2)	1.76(2)	2.663(2)	173(2)
	1.219(4)	1.219(4)	2.4256(19)	169(3)

Table S4. Dihedral angles between phenol ring and benzopyrone ring of non-equivalent HES species in HESTEA polymorphs and reported crystal structures deposited in CSD.

Refcode	Dihedral angles	Refcode	Dihedral angles
HESTEA-*α*	75.51	LAVLAY	19.85
	89.06	LAVLEC	86.18
HESTEA-*β*	83.56	LAVLIG	46.27
	85.05	RUWHEX	7.11
HESTEA-*γ*	77.3		10.64
FOYTOC	3.69	RUWHIB	4.69
IJIWAC	40.17		

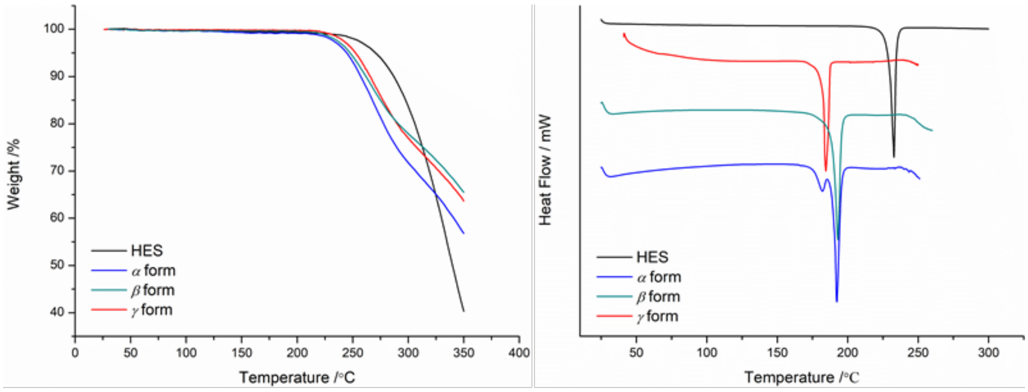

(a) (b)

Figure S3. TGA and DSC curves of HESTEA-*α*, -*β* and -*γ*.

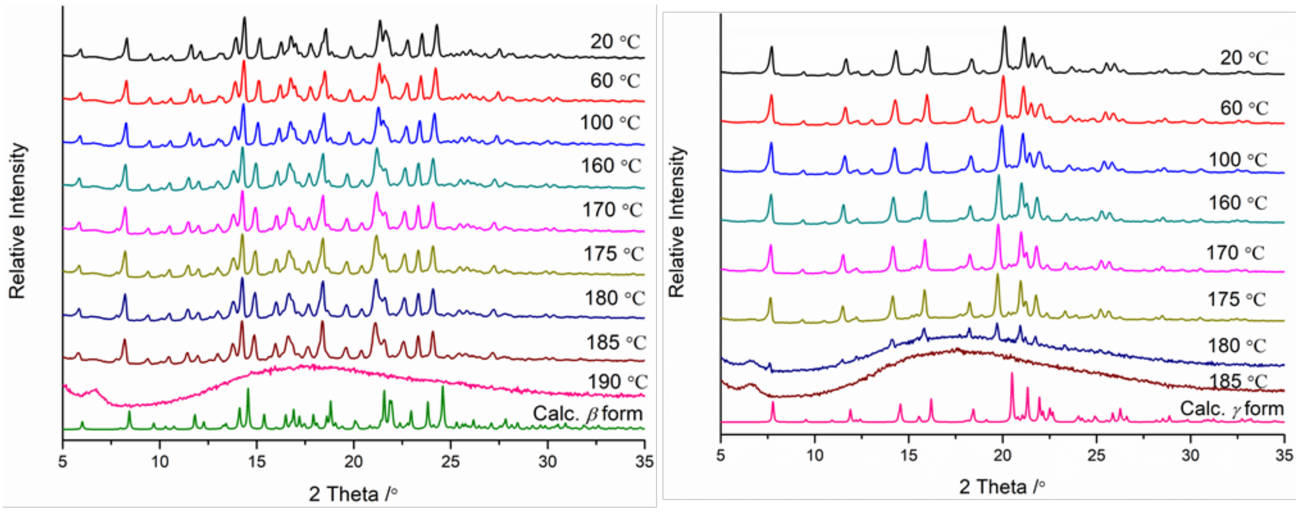

(a) (b)

Figure S4. Variable temperature PXRD of (a) HESTEA-β and (b) HESTEA-γ (there is peak shift due to thermal expansion as ICCs were collected at 100K).

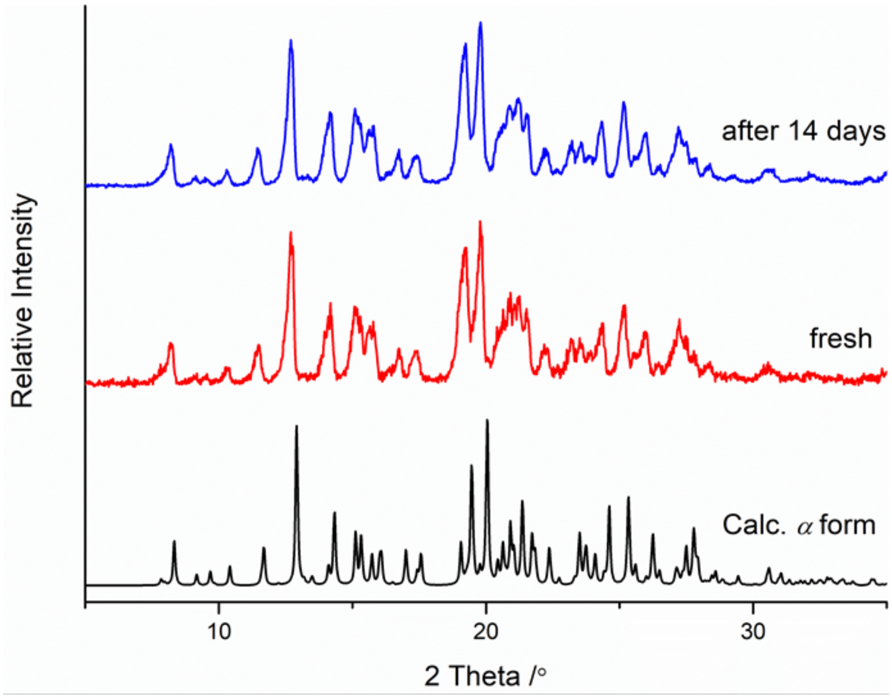

(a)

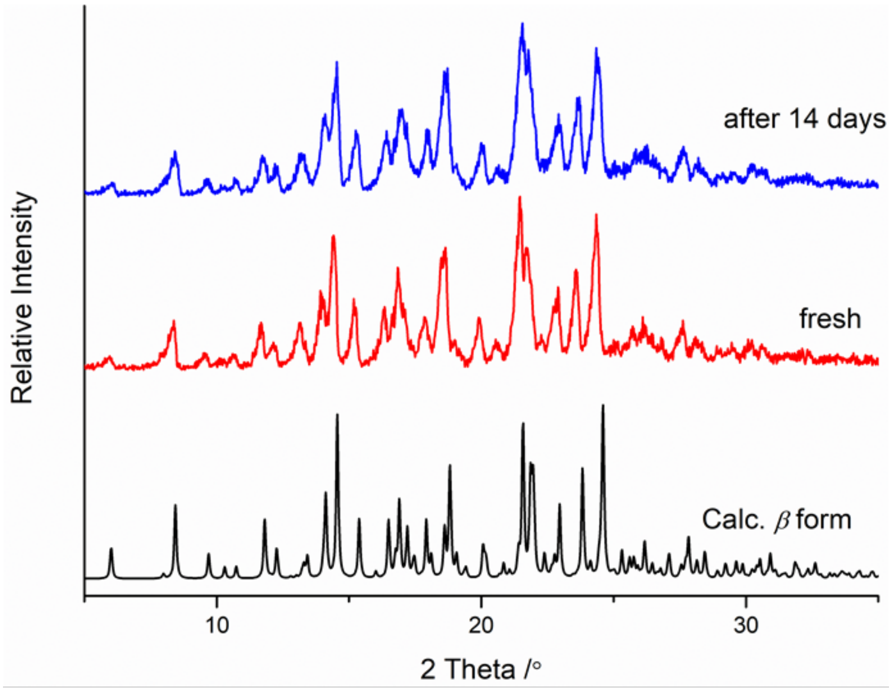

after 14 days
fresh
Calc. β form
Relative Intensity
10
20
30
2 Theta /°

(b)

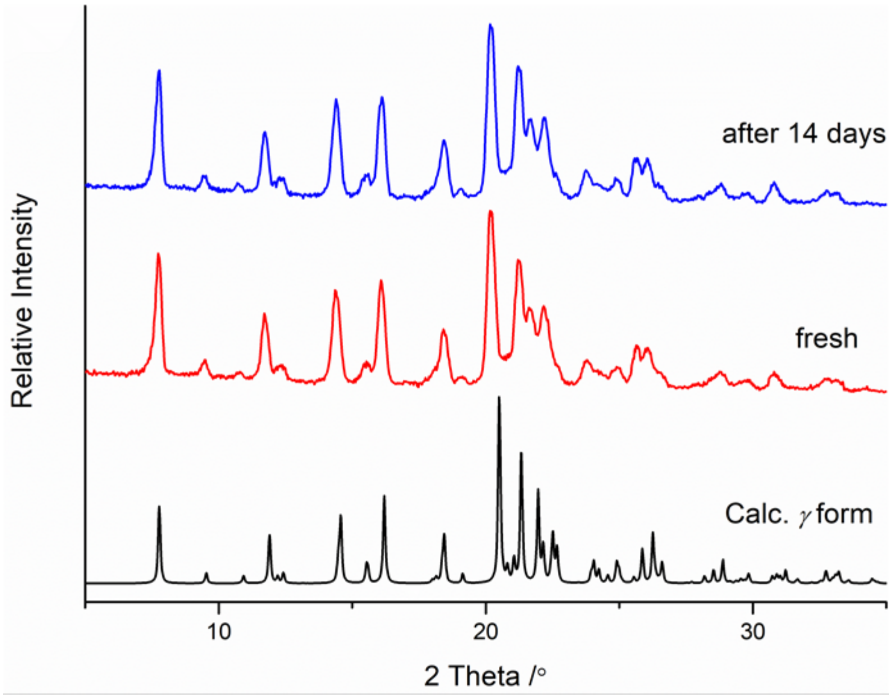

after 14 days
fresh
Calc. γ form
Relative Intensity
10
20
30
2 Theta /°

(c)

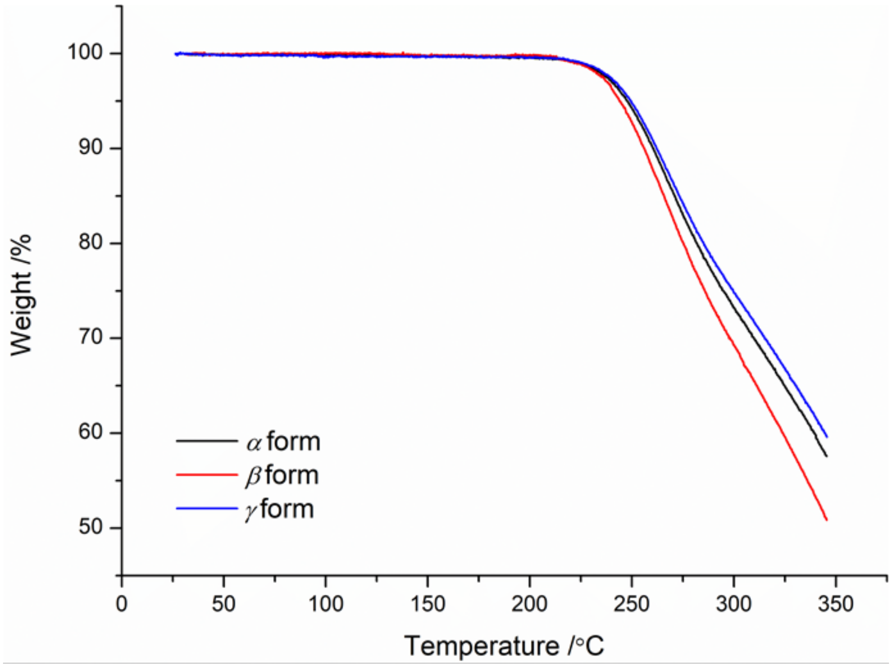

(d)

Figure S5. PXRD patterns and TGA spectra of HESTEA-*α*, -*β* and -*γ* after 14 days in accelerated stability test (there is peak shift due to thermal expansion as ICCs were collected at 100K).

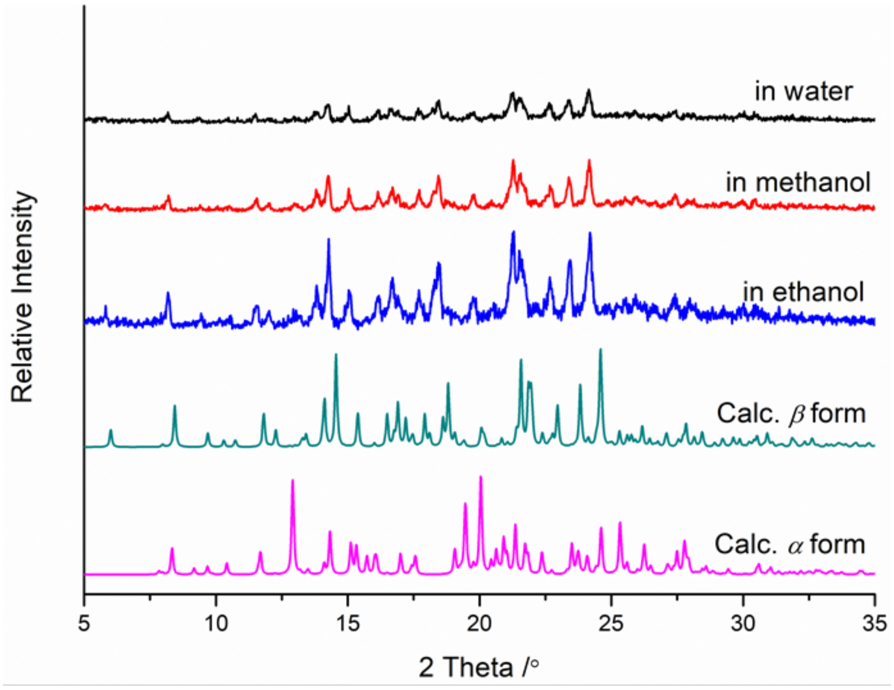

(a)

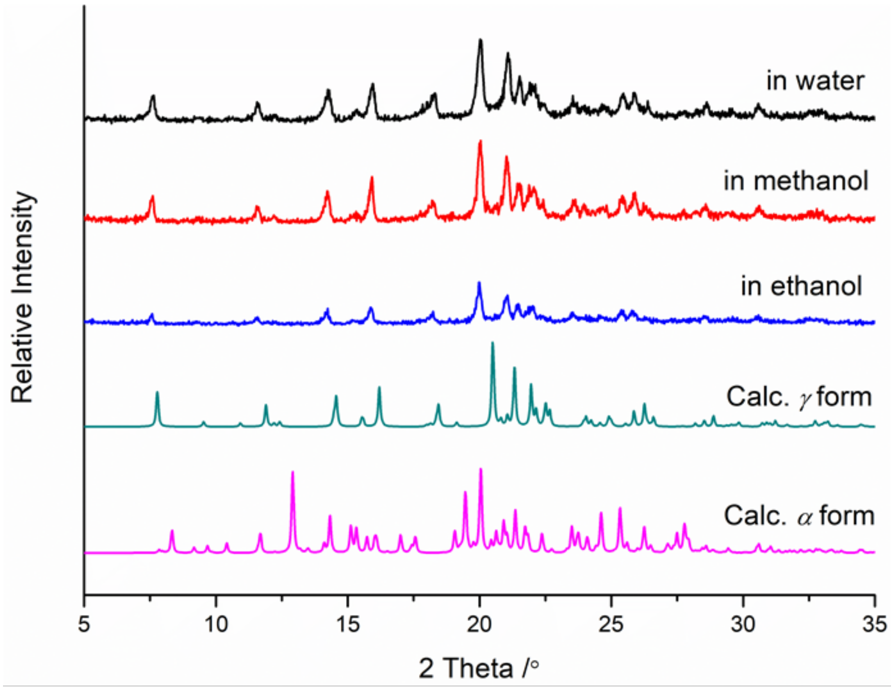

(b)

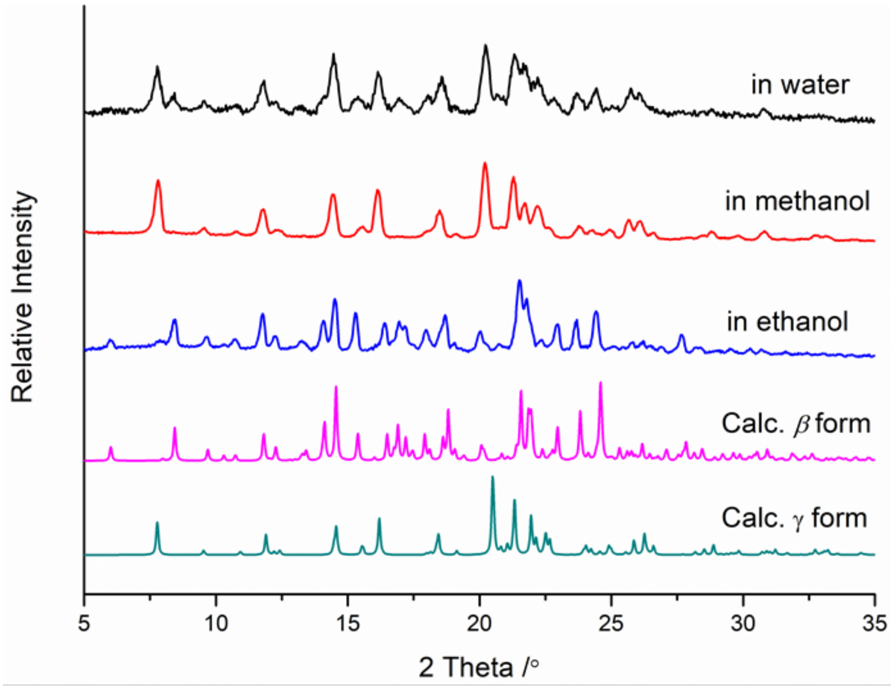

(c)

Figure S6. PXRD of slurry conversion experiments (a) HESTEA-$\alpha + \beta$ (b) HESTEA-$\alpha + \gamma$ (c) HESTEA-$\beta + \gamma$ (there is peak shift due to thermal expansion as ICCs were collected at 100K).

Reference

1. Allu, S.; Bolla, G.; Tothadi, S.; Nangia, A. Supramolecular Synthons in Bumetanide Cocrystals and Ternary Products. *Cryst. Growth Des.* **2017,** 17, 4225-4236.
2. Allu, S.; Bolla, G.; Tothadi, S.; Nangia, A. K. Novel Pharmaceutical Cocrystals and Salts of Bumetanide. *Cryst. Growth Des.* **2019,** 20, 793-803.
3. Gunnam, A.; Suresh, K.; Nangia, A. Salts and Salt Cocrystals of the Antibacterial Drug Pefloxacin. *Cryst. Growth Des.* **2018,** 18, 2824-2835.
4. Petruševski, G.; Naumov, P.; Jovanovski, G.; Ng, S. W. Unprecedented sodium–oxygen clusters in the solid-state structure of trisodium hydrogentetravalproate monohydrate: A model for the physiological activity of the anticonvulsant drug Epilim®. *Inorg. Chem. Commun.* **2008,** 11, 81-84.
5. Perumalla, S. R.; Sun, C. C. Design and synthesis of solid state structures with conjugate acid–base pair interactions. *CrystEngComm.* **2012,** 14, 3851–3853.
6. Perumalla, S. R.; Sun, C. C. Improved solid-state stability of salts by cocrystallization between conjugate acid–base pairs. *CrystEngComm.* **2013,** 15, 5756-5759.
7. Umeda, Y.; Nagase, H.; Makimmura, M.; Tomono, K.; ShiroHIRO, M.; Ueda, H. Crystal Structure of a 2:1 Complex of Indomethacin and Lidocaine. *Anal. Sci.* **2007,** 23, x15-x16.
8. Búdová, M.; Skořepová, E.; Jan, Č. Sodium Aspirin Salts: Crystallization and Characterization. *Cryst. Growth Des.* **2018,** 18, 5287-5294.
9. Sa, R.; Zhang, Y.; Deng, Y.; Huang, Y.; Zhang, M.; Lou, B. Novel Salt Cocrystal of Chrysin with Berberine: Preparation, Characterization, and Oral Bioavailability. *Cryst. Growth Des.* **2018,** 18, 4724-4730.
10. Harrison, W. T.; Yathirajan, H. S.; Bindya, S.; Anilkumar, H. G.; Devaraju. Escitalopram oxalate: co-existence of oxalate dianions and oxalic acid molecules in the same crystal. *Acta Crystallogr. C.* **2007,** 63, o129-o131.
11. Cherukuvada, S.; Bolla, G.; Sikligar, K.; Nangia, A. 4-Aminosalicylic Acid Adducts. *Cryst. Growth Des.* **2013,** 13, 1551-1557.
